# Nut consumption and incidence of seven cardiovascular diseases

**DOI:** 10.1136/heartjnl-2017-312819

**Published:** 2018-04-16

**Authors:** Susanna C Larsson, Nikola Drca, Martin Björck, Magnus Bäck, Alicja Wolk

**Affiliations:** 1 Unit of Nutritional Epidemiology, Institute of Environmental Medicine, Karolinska Institutet, Stockholm, Sweden; 2 Department of Cardiology, Karolinska University Hospital, Karolinska Institutet, Stockholm, Sweden; 3 Department of Surgical Sciences, Uppsala University, Uppsala, Sweden; 4 Divison of Valvular and Coronary Disease, Karolinska University Hospital, Stockholm, Sweden; 5 Center for Molecular Medicine, Department of Medicine, Karolinska Institutet, Stockholm, Sweden

**Keywords:** atrial fibrillation, heart failure, aortic aneurysm, aortic stenosis, acute myocardial infarction

## Abstract

**Background:**

Nut consumption has been found to be inversely associated with cardiovascular disease mortality, but the association between nut consumption and incidence of specific cardiovascular diseases is unclear. We examined the association between nut consumption and incidence of seven cardiovascular diseases.

**Methods:**

This prospective study included 61 364 Swedish adults who had completed a Food Frequency Questionnaire and were followed up for 17 years through linkage with the Swedish National Patient and Death Registers.

**Results:**

Nut consumption was inversely associated with risk of myocardial infarction, heart failure, atrial fibrillation and abdominal aortic aneurysm in the age-adjusted and sex-adjusted analysis. However, adjustment for multiple risk factors attenuated these associations and only a linear, dose–response, association with atrial fibrillation (p_trend_=0.004) and a non-linear association (p_non-linearity_=0.003) with heart failure remained. Compared with no consumption of nuts, the multivariable HRs (95% CI) of atrial fibrillation across categories of nut consumption were 0.97 (0.93 to 1.02) for 1–3 times/month, 0.88 (0.79 to 0.99) for 1–2 times/week and 0.82 (0.68 to 0.99) for ≥3 times/week. For heart failure, the corresponding HRs (95% CI) were 0.87 (0.80 to 0.94), 0.80 (0.67 to 0.97) and 0.98 (0.76 to 1.27). Nut consumption was not associated with risk of aortic valve stenosis, ischaemic stroke or intracerebral haemorrhage.

**Conclusions:**

These findings suggest that nut consumption or factors associated with this nutritional behaviour may play a role in reducing the risk of atrial fibrillation and possibly heart failure.

**Trial registration number:**

NCT01127711 and NCT01127698; Results.

## Introduction

Nuts are rich sources of unsaturated fatty acids, protein, fibre, minerals (eg, magnesium, potassium and zinc), vitamin E, folate and other bioactive compounds such as phenolics and phytosterols.^1^ Nut consumption may influence cardiovascular health by improving blood lipid levels^1 2^ and endothelial function,^3^ reducing the risk of weight gain,^4^ and via antioxidant and anti-inflammatory effects.^1^ Meta-analyses of prospective studies have shown that nut consumption is inversely associated with death from cardiovascular disease (CVD), total coronary heart disease and total stroke.^5–7^ Data from prospective studies on nut consumption in relation to incidence of specific CVD outcomes, such as myocardial infarction,^8 9^ heart failure,^10 11^ atrial fibrillation,^12^ aortic valve stenosis and abdominal aortic aneurysm are scarce.

To further evaluate the potential role of nut consumption for prevention of CVD and in particular to elucidate whether there are CVD outcome-specific associations, we used data from two population-based cohort studies of Swedish adults. We investigated the association of nut consumption with incidence of seven CVD outcomes.

## Methods

### Study population

The study population for the present study was participants of the Cohort of Swedish Men and the Swedish Mammography Cohort. In the autumn of 1997, 48 850 men (born 1918–1952) and 39 227 women (born 1914–1948) from three Swedish counties completed a questionnaire that sought information on diet, lifestyle and other risk factors for chronic diseases. For this analysis, we excluded participants with a missing or an erroneous personal identity number (a unique number assigned to each Swedish resident at birth), those with prevalent CVD or cancer recorded in the Swedish registers, those with extreme energy intake (ie, 3 SD from the log_e_-transformed mean energy intake among men and women separately), and those who did not answer the question about nut consumption (online supplementary figure S1). This left 61 364 participants (32 911 men and 28 453 women), aged 45–83 years, for analyses.

10.1136/heartjnl-2017-312819.supp1 Supplementary file 1

### Exposure assessment

Participants completed a baseline questionnaire that inquired about diet, smoking status and history, weight, height, physical activity, alcohol consumption, family history of myocardial infarction, aspirin use and history of diabetes, hypertension and hypercholesterolaemia. We defined a history of diabetes based on self-report or a previous (before baseline) diagnosis of type 2 diabetes recorded in the Swedish National Patient or Diabetes Registers. The average consumption of nuts/almonds (hereafter referred to as nuts) and other foods and food items during the previous year was assessed with a 96-item Food Frequency Questionnaire. Nuts did not include coconut or chestnut. Participants could choose from eight predefined frequency categories of nut consumption (none, 1–3 times/month, 1–2 times/week, 3–4 times/week, 5–6, times/week, 1 time/day, 2 times/day and ≥3 times/day).

### Case ascertainment

Cases of CVD were ascertained through linkage with the Swedish National Patient and Cause of Death Registers (using the unique personal identity number assigned to each Swedish resident) and classified according to the International Classification of Diseases, 10th Revision codes. The endpoints in the present study were acute myocardial infarction (I21), heart failure (I50 and I11.0), atrial fibrillation (I48), aortic valve stenosis (I35.0 and I35.2), abdominal aortic aneurysm (I71.3 and I71.4), ischaemic stroke (I63)and intracerebral haemorrhage (I61). We further divided myocardial infarction into non-fatal and fatal events. Fatal myocardial infarction was defined as death from myocardial infarction within 28 days from the event. The validity of diagnoses in the Swedish National Patient Register is high, with a positive predictive value close to 100% for some diseases (eg, myocardial infarction and atrial fibrillation) and >85% for many other diagnoses.^13^

### Statistical analysis

Person-time of follow-up for each participant was calculated from 1 January 1998 to the date of diagnosis of each endpoint, date of death or 31 December 2014, whichever occurred first. Participants were classified into one of the following categories of nut consumption: none, 1–3 times/month, 1–2 times/week and ≥3 times/week. HRs with their 95% CIs for each endpoint by categories of nut consumption were estimated using Cox proportional hazards models with age as the time scale and stratified by sex. The first multivariable model was additionally adjusted for education (less than high school, high school or university), family history of myocardial infarction before 60 years of age (yes/no), smoking (never, past <20 pack-years, past ≥20 pack-years, current <20 pack-years or current ≥20 pack-years), walking/bicycling (almost never, <20 min/day, 20–40 min/day or >40 min/day), exercise (<1 hour/week, 1–2 hours/week, 3–4 hours/week or ≥5 hours/week), aspirin use (never, 1–6 tablets/week or ≥7 tablets/week) and consumption of alcohol (never drinkers, past drinkers or current drinkers of <1 drink/week, 1–6 drinks/week, 7–14 drinks/week, 14–21 drinks/week or >21 drinks/week), fruits (quintiles), vegetables (quintiles) and total energy (kcal/day; continuous). A second multivariable model further adjusted for potential intermediates of the association between nut consumption and CVD, including body mass index (weight divided by the square of height (kg/m^2^): <22.5, 22.5–24.9, 25.0–29.9 or ≥30.0), history of diabetes (yes/no), history of hypertension (yes/no) and history of hypercholesterolaemia (yes/no). A separate missing category for each covariable was used to handle missing data (<5% missing). We considered adjustment for other foods and beverages, including whole grain foods, low-fat dairy products, fish, processed and unprocessed red meat, chocolate, sweets, and sweetened beverages. Because adjustment for these foods did not alter the results, they were not included in the multivariable model. The proportionality assumption was verified using Schoenfeld residuals.

To test for a linear trend, we assigned the midpoint to each category of nut consumption and modelled this variable as a continuous variable. In a sensitivity analysis, we excluded individuals with a history of diabetes at baseline as they may have changed their nut consumption after the diagnosis. Furthermore, we conducted a sensitivity analysis with follow-up time restricted to 10 years. The rationale for this is that the impact of regression dilution bias due to changes in nut consumption would be less with shorter follow-up. We also conducted a sensitivity analysis excluding the first 2 years of follow-up to evaluate potential bias due to reverse causality. All statistical tests were two sided and p values below 0.05 were considered statistically significant. The analyses were conducted using SAS V. 9.4 and Stata V.14.2.

## Results

Characteristics of study participants by categories of nut consumption are shown in table 1. Compared with non-consumers of nuts, those with high nut consumption were more likely to have a postsecondary education and to be physically active, but were less likely to be current smokers and to have a history of hypertension. Those with high nut consumption were also, on average, younger and had lower body mass index and higher consumption of alcohol, fruits and vegetables compared with non-consumers of nut. There was a U-shaped association between nut consumption and history of diabetes.

**Table 1:** Baseline characteristics of participants according to nut consumption

Characteristic*	Frequency of nut consumption			
	None (n=32 334)	1–3/month (n=24 707)	1–2/week (n=3315)	≥3/week (n=1008)
Age (years)	60.1	57.5	56.7	56.6
Postsecondary education (%)	16.3	23.4	33.9	36.8
Family history of myocardial infarction (%)	15.1	14.9	15.9	13.8
Current smoker (%)	25.7	22.0	22.8	22.6
Body mass index (kg/m^2^)	25.6	25.1	24.9	24.6
Walking/bicycling ≥40 min/day (%)	39.2	38.2	38.2	46.9
Exercise ≥2 hours/week (%)	63.1	64.5	67.9	70.1
Diabetes (%)	5.3	3.5	3.4	6.0
Hypertension (%)	20.9	18.6	16.7	16.9
Hypercholesterolaemia (%)	11.0	10.2	9.8	10.9
Aspirin use ≥7 tablets/week (%)	6.1	6.3	6.3	5.5
Alcohol (drinks/week)†	6.6	6.9	8.8	9.9
Total energy intake (kcal/day)	2200	2300	2500	2700
Fruit consumption (servings/day)	1.7	1.9	2.1	2.5
Vegetable consumption (servings/day)	2.8	3.1	3.5	4.0

*Standardised to the age distribution of the study population at baseline. Values are means if not otherwise indicated.

†Among current drinkers (1 drink equals 12 g alcohol).

The number of incident CVD events ascertained during 17 years of follow-up of 61 364 participants was 4983 for myocardial infarction (4066 non-fatal and 917 fatal), 3160 for heart failure, 7550 for atrial fibrillation, 972 for aortic valve stenosis, 983 for abdominal aortic aneurysm, 3782 for ischaemic stroke and 543 for intracerebral haemorrhage.

**Figure 1:**
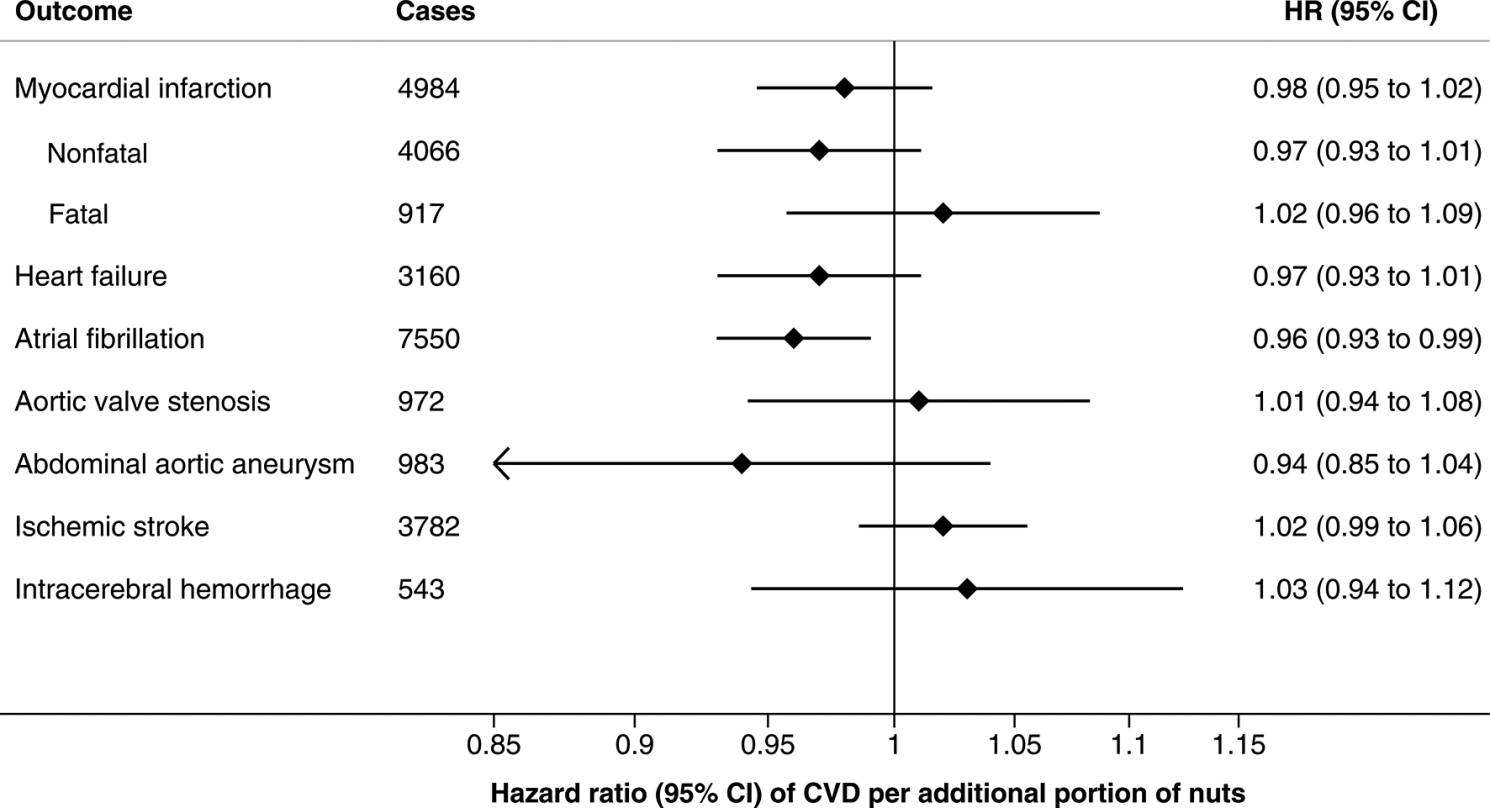
Multivariable HRs (95% CI) of incident cardiovascular disease (CVD) per additional portion of nuts per week. HRs were estimated by Cox proportional hazards models with age as the time scale and stratified by sex, and adjusted for education (less than high school, high school or university), family history of myocardial infarction before 60 years of age (yes/no), smoking (never, past <20 pack-years, past ≥20 pack-years, current <20 pack-years or current ≥20 pack-years), walking/bicycling (almost never, <20 min/day, 20–40 min/day or >40 min/day), exercise (<1 hour/week, 1–2 hours/week, 3–4 hours/week or ≥5 hours/week), aspirin use (never, 1–6 tablets/week or ≥7 tablets/week) and consumption of alcohol (never drinkers, past drinkers or current drinkers of <1 drink/week, 1–6 drinks/week, 7–14 drinks/week, 14–21 drinks/week or >21 drinks/week), fruits (quintiles), vegetables (quintiles), total energy (kcal/day; continuous), body mass index (<22.5, 22.5–24.9, 25.0–29.9or ≥30.0 kg/m^2^), history of diabetes (yes/no), history of hypertension (yes/no) and history of hypercholesterolaemia (yes/no).

In the age-adjusted and sex-adjusted analysis, nut consumption was inversely associated with risk of total and non-fatal myocardial infarction, heart failure, atrial fibrillation and abdominal aortic aneurysm (table 2). Adjustment for multiple risk factors attenuated these associations and only a linear, dose–response, association with atrial fibrillation (p_trend_=0.004) and a non-linear association (p_non-linearity_=0.003) with heart failure remained in the fully adjusted model (multivariable model 2) (table 2). The main confounder was education in the analysis of myocardial infarction and fruit consumption in the analysis of abdominal aortic aneurysm. Compared with no nut consumption, the HRs of atrial fibrillation across categories of nut consumption were 0.97 (95% CI 0.93 to 1.02) for 1–3 times/month, 0.88 (95% CI 0.79 to 0.99) for 1–2 times/week and 0.82 (95% CI 0.68 to 0.99) for ≥3 times/week. For heart failure, the corresponding HRs were 0.87 (95% CI 0.80 to 0.94), 0.80 (95% CI 0.67 to 0.97) and 0.98 (95% CI 0.76 to 1.27). Nut consumption was not associated with risk of aortic valve stenosis, ischaemic stroke or intracerebral haemorrhage (table 2 and figure 1). Each additional portion of nuts per week was associated with a 4% reduction in risk of atrial fibrillation, but was not significantly associated with the other CVD outcomes (figure 1).

**Table 2:** HR (95% CI) of incident CVD according to nut consumption in Swedish adults, 1998–2014

Outcome	Frequency of nut consumption				P_trend_	
	None	1–3/month	1–2/week	≥3/week	Linear	Non-linear
Myocardial infarction						
Cases (n=4983)	2962	1734	213	74		
Age-adjusted and sex-adjusted model*	1.00 (reference)	0.88 (0.83 to 0.93)	0.79 (0.69 to 0.91)	0.77 (0.61 to 0.96)	<0.001	<0.001
Multivariable model 1†	1.00 (reference)	0.96 (0.90 to 1.02)	0.88 (0.77 to 1.02)	0.86 (0.68 to 1.09)	0.04	0.10
Multivariable model 2†‡	1.00 (reference)	0.98 (0.92 to 1.04)	0.91 (0.79 to 1.05)	0.88 (0.70 to 1.11)	0.12	0.26
Non-fatal myocardial infarction						
Cases (n=4066)	2368	1466	182	51		
Age-adjusted and sex-adjusted model*	1.00 (reference)	0.92 (0.86 to 0.98)	0.83 (0.71 to 0.96)	0.66 (0.50 to 0.88)	<0.001	0.01
Multivariable model 1†	1.00 (reference)	0.99 (0.92 to 1.06)	0.92 (0.79 to 1.08)	0.75 (0.57 to 0.99)	0.03	0.31
Multivariable model 2†‡	1.00 (reference)	1.01 (0.95 to 1.08)	0.95 (0.82 to 1.11)	0.76 (0.58 to 1.01)	0.09	0.26
Fatal myocardial infarction						
Cases (n=917)	595	268	31	23		
Age-adjusted and sex-adjusted model*	1.00 (reference)	0.73 (0.63 to 0.84)	0.63 (0.44 to 0.91)	1.19 (0.78 to 0.80)	0.14	<0.001
Multivariable model 1†	1.00 (reference)	0.82 (0.71 to 0.95)	0.72 (0.50 to 1.05)	1.32 (0.86 to 2.01)	0.78	0.009
Multivariable model 2†‡	1.00 (reference)	0.85 (0.73 to 0.99)	0.75 (0.52 to 1.09)	1.34 (0.88 to 2.05)	0.99	0.03
Heart failure						
Cases (n=3160)	2034	950	114	62		
Age-adjusted and sex-adjusted model*	1.00 (reference)	0.78 (0.72 to 0.84)	0.72 (0.60 to 0.87)	0.94 (0.73 to 1.21)	0.001	<0.001
Multivariable model 1†	1.00 (reference)	0.83 (0.77 to 0.90)	0.76 (0.63 to 0.92)	0.94 (0.73 to 1.22)	0.01	<0.001
Multivariable model 2†‡	1.00 (reference)	0.87 (0.80 to 0.94)	0.80 (0.67 to 0.97)	0.98 (0.76 to 1.27)	0.07	0.003
Atrial fibrillation						
Cases (n=7550)	4372	2737	328	113		
Age-adjusted and sex-adjusted model*	1.00 (reference)	0.97 (0.92 to 1.01)	0.88 (0.79 to 0.99)	0.87 (0.67 to 0.89)	0.002	0.18
Multivariable model 1†	1.00 (reference)	0.95 (0.91 to 1.00)	0.85 (0.76 to 0.95)	0.79 (0.65 to 0.95)	<0.001	0.06
Multivariable model 2†‡	1.00 (reference)	0.97 (0.93 to 1.02)	0.88 (0.79 to 0.99)	0.82 (0.68 to 0.99)	0.004	0.24
Aortic valve stenosis						
Cases (n=972)	561	356	41	14		
Age-adjusted and sex-adjusted model*	1.00 (reference)	1.01 (0.89 to 1.16)	0.90 (0.66 to 1.24)	0.78 (0.46 to 1.33)	0.29	0.26
Multivariable model 1†	1.00 (reference)	1.06 (0.92 to 1.21)	0.95 (0.69 to 1.31)	0.83 (0.48 to 1.41)	0.61	0.25
Multivariable model 2†‡	1.00 (reference)	1.09 (0.95 to 1.26)	1.00 (0.72 to 1.37)	0.87 (0.51 to 1.41)	0.89	0.20
Abdominal aortic aneurysm						
Cases (n=983)	608	324	42	9		
Age-adjusted and sex-adjusted model*	1.00 (reference)	0.84 (0.74 to 0.97)	0.82 (0.60 to 1.12)	0.49 (0.25 to 0.94)	0.03	0.12
Multivariable model 1†	1.00 (reference)	0.93 (0.81 to 1.07)	0.90 (0.65 to 1.24)	0.56 (0.29 to 1.09)	0.06	0.47
Multivariable model 2†‡	1.00 (reference)	0.94 (0.82 to 1.08)	0.92 (0.67 to 1.26)	0.58 (0.30 to 1.13)	0.89	0.53
Ischaemic stroke						
Cases (n=3782)	2202	1335	180	65		
Age-adjusted and sex-adjusted model*	1.00 (reference)	0.95 (0.89 to 1.02)	1.00 (0.86 to 1.16)	0.93 (0.73 to 1.19)	0.42	0.24
Multivariable model 1†	1.00 (reference)	1.01 (0.94 to 1.08)	1.09 (0.93 to 1.27)	1.01 (0.79 to 1.30)	0.54	0.80
Multivariable model 2†‡	1.00 (reference)	1.04 (0.97 to 1.11)	1.12 (0.96 to 1.31)	1.03 (0.80 to 1.32)	0.29	0.49
Intracerebral haemorrhage						
Cases (n=543)	306	205	22	10		
Age-adjusted and sex-adjusted model*	1.00 (reference)	1.01 (0.84 to 1.21)	0.83 (0.54 to 1.28)	1.04 (0.55 to 1.95)	0.78	0.28
Multivariable model 1†	1.00 (reference)	1.04 (0.86 to 1.25)	0.84 (0.54 to 1.30)	1.00 (0.53 to 1.90)	0.79	0.27
Multivariable model 2†‡	1.00 (reference)	1.04 (0.86 to 1.25)	0.84 (0.54 to 1.30)	1.00 (0.53 to 1.89)	0.77	0.29

*HRs were estimated by Cox proportional hazards models with age as the time scale and stratified by sex.

†HRs were estimated by Cox proportional hazards models with age as the time scale and stratified by sex, and adjusted for education (less than high school, high school or university), family history of myocardial infarction before 60 years of age (yes/no), smoking (never, past <20 pack-years, past ≥20 pack-years, current <20 pack-years or current ≥20 pack-years), walking/bicycling (almost never,<20 min/day, 20–40 min/day or >40 min/day), exercise (<1 hour/week, 1–2 hours/week, 3–4 hours/week or ≥5 hours/week), aspirin use (never, 1–6 tablets/week or ≥7 tablets/week) and consumption of alcohol (never drinkers, past drinkers or current drinkers of <1 drink/week, 1–6 drinks/week, 7–14 drinks/week, 14–21 drinks/week or >21 drinks/week), fruits (quintiles), vegetables (quintiles) and total energy (kcal/day; continuous).

‡HRs were adjusted for the same variables as in model 1 and further for potential intermediates of the nut–CVD relationship, including body mass index (<22.5, 22.5–24.9, 25.0–29.9 or ≥30.0 kg/m^2^), history of diabetes (yes/no), history of hypertension (yes/no) and history of hypercholesterolaemia (yes/no).

CVD, cardiovascular disease.

In sensitivity analyses, the results for all CVD outcomes were similar after exclusion of individuals with a history of diabetes (online supplementary table S1) or when the follow-up time was restricted to 10 years (online supplementary table S2). Likewise, omitting the first 2 years of follow-up did not change the results appreciably (online supplementary table S3).

In analyses stratified by sex, nut consumption was inversely associated with risk of atrial fibrillation in both men and women but results did not attain statistical significance (online supplementary figure S2). A borderline statistically significant association between nut consumption and risk of abdominal aortic aneurysm was observed in women but not in men (p-interaction by sex=0.02) (online supplementary figure S2).

## Discussion

In this large prospective study, we observed a previously unrecognised inverse association between nut consumption and incident atrial fibrillation, which remained after adjustment for multiple risk factors. Nut consumption ≥3 times/week was associated with an 18% reduced risk of atrial fibrillation. We also observed inverse associations of nut consumption with risk of total and non-fatal myocardial infarction, heart failure and abdominal aortic aneurysm after adjustment for age and sex only. However, adjustment for other risk factors attenuated these associations and only a non-linear association with heart failure persisted. Nut consumption was not associated with risk of fatal myocardial infarction, aortic valve stenosis, ischaemic stroke or intracerebral haemorrhage.

Few prospective studies and randomised trials have reported on the association between nut consumption and incidence of specific CVD outcomes, and the findings are inconsistent. Nut consumption was not associated with risk of non-fatal myocardial infarction,^9^ heart failure^11^ or atrial fibrillation^12^ in the Physicians’ Health Study or with heart failure in the Atherosclerosis Risk in Community Study.^10^ The Adventist Health Study showed an inverse association between nut consumption and risk of non-fatal myocardial infarction but the number of cases was limited (n=134).^8^ The PREvención con DIeta MEDiterránea (PREDIMED) randomised controlled trial, a Mediterranean diet supplemented with mixed nuts, compared with a control diet (reduced fat diet), did not significantly reduce the risk of the non-primary endpoints of myocardial infarction (n=69 total cases in the nut and control groups; HR 0.74, 95% CI 0.46 to 1.19),^14^ heart failure (n=65 cases; HR 0.92, 95% CI 0.56 to 1.49)^15^ or atrial fibrillation (n=181 cases; HR 0.89, 95% CI 0.65 to 1.20).^16^ However, with such small incidence numbers, there is a high risk of statistical type 2 error. While results on nut consumption and incidence of myocardial infarction are inconclusive, the overall evidence indicates that nut consumption is inversely associated with mortality from CVD,^5–7^ in particular coronary heart disease.^5^ In a meta-analysis of 11 prospective studies, the HR of coronary heart disease was 0.76 (95% CI 0.69 to 0.84) for the highest versus lowest category of nut consumption.^5^

The association between nut consumption and abdominal aortic aneurysm was examined in a retrospective cohort of 3.1 million US adults who were evaluated by ultrasound imaging for the presence of abdominal aortic aneurysm.^17^ In that large study, consumption of nuts >3 times/week was associated with lower odds of abdominal aortic aneurysm prevalence (OR 0.90, 95% CI 0.89 to 0.93).^17^ In the present study, an inverse association of nut consumption with incidence of abdominal aortic aneurysm was observed after adjustment for age and sex only, but the association did not persist after adjustment for other factors related to risk of abdominal aortic aneurysm in the these cohorts, such as smoking, alcohol and fruit consumption.^18–20^ The discrepancy in results could be explained by the lower number of incident cases in this study, or by reverse causation bias or residual confounding in the US study.

Aortic stenosis shares several traditional cardiovascular risk factors with coronary and cerebrovascular disease but the association of dietary factors with this valvular heart disease has remained unexplored. To the best of our knowledge, no previous study has investigated the possible association between nut consumption and risk of aortic valve stenosis, which could not be verified in the present study.

The overall evidence from this study along with previous cohort studies indicates that nut consumption is not associated with a reduced risk of ischaemic^21–25^ or haemorrhagic^21 22 24 25^ stroke in European and US adults. If anything, a positive association has been reported.^21 23^ In contrast, a prospective study of Chinese adults found a significant lower risk of mortality from ischaemic stroke and intracerebral haemorrhage among those with as little as 1.45 g/day or more of nuts (two highest quintiles).^25^ Although observational data provide little support (except for in Chinese adults) that nut consumption lowers the risk of stroke, the PREDIMED trial showed that individuals randomised to a Mediterranean diet supplemented with nuts experienced a 46% reduced risk of stroke (HR 0.54, 95% CI 0.35 to 0.84) compared with the control group (advise to follow a low-fat diet).^14^ The reason for the inconsistent findings is unclear but might be related to the type and amount of nuts consumed. In the PREDIMED trial, those randomised to the nut group received 30 g/day of mixed nuts (walnuts, almonds and hazelnuts). In the prospective observational studies, however, nut consumption was lower and likely a mixture of unsalted and salted nuts.

The observed inverse associations of nut consumption with risk of atrial fibrillation (linear dose–response association) and heart failure (non-linear association) might in part be mediated by weight changes as previous prospective studies have shown that high nut consumption is associated with significantly less weight gain during follow-up.^4^ In the present cohorts, overweight and obesity are strongly associated with increased risk of atrial fibrillation^26^ and heart failure^27^ but also with aortic valve stenosis^28^ and abdominal aortic aneurysm (abdominal obesity only).^29^ The reduced risk of heart failure associated with moderate but not high nut consumption might be related to that high nut consumption increases rather than decreases body weight. Nuts may also beneficially influence cardiovascular health through anti-inflammatory and antioxidant effects,^1^ improvement of endothelial function^3^ and reduction of total and low-density lipoprotein cholesterol levels.^1 2^ However, the effect of nut intake on cholesterol appears to be non-linear with an effect primarily seen at intakes of at least 60 g/day.^2^ Nut consumption in this study population may have been too low to have a meaningful impact on cholesterol levels. Atrial fibrosis has been identified as a fundamental structural change responsible for the perpetuation of atrial fibrillation.^30^ Whether the anti-inflammatory and antioxidant effects of nuts could have a preventive effect on atrial fibrosis is to the best of our knowledge unknown.

Strengths of this study include the large sample size, the large number of incident cases for most CVD endpoints and the assessment of nut consumption at baseline in relation to risk of multiple CVD endpoints in the same population. Another major strength is the objective and virtually complete case ascertainment accomplished by record linkage with the unique personal identity number used in all the registries.

Regression dilution bias due to measurement error in the assessment of nut consumption at baseline and changes in nut consumption during follow-up may have attenuated the results. Attenuation of the risk estimates due to changes in nut consumption is expected to increase with longer follow-up. We conducted a sensitivity analysis restricted to the first 10 years of follow-up and observed somewhat stronger associations (HRs further away from the null) but with less precision in the estimates because of fewer incident cases. Because this was an observational study, we cannot rule out the possibility that the observed associations are due to unmeasured or residual confounding. Participants who frequently consumed nuts were more likely to adhere to healthy lifestyle behaviours and had fewer other risk factors for CVD compared with non-consumers of nuts. We cannot rule out residual confounding by income and occupation because we could not adjust for these potential confounders. Another potential concern is reverse causation bias, which was addressed by excluding the first 2 years of follow-up. Results were similar in this sensitivity analysis. Since multiple CVD outcomes were analysed, we cannot exclude the possibility of chance findings. Finally, as this study population comprised Swedish middle-aged and older adults only, our findings might not be generalisable to other populations with potentially other proportions and types of nuts consumed.

In conclusion, results from this large prospective study suggest that nut consumption or factors associated with this nutritional behaviour may play a role in reducing the risk of atrial fibrillation and possibly heart failure. Since only a small proportion of this population had moderate (about 5%) or high (<2%) nut consumption, even a small increase in nut consumption may have large potential to lead to a reduction in incidence of atrial fibrillation and heart failure in this population.

**Key messages**

**What is already known on this subject?**

- Previous studies have found that nut consumption is inversely associated with cardiovascular disease mortality.

**What might this study add?**

- This study shows that consumption of nuts ≥3 times/week is associated with an 18% reduced risk of atrial fibrillation. Moderate (up to 1–2 times/week) but not high consumption of nuts was associated with a reduced risk of heart failure.

**How might this impact on clinical practice?**

- These findings suggest that nut consumption may play a role in reducing the risk of atrial fibrillation and possibly heart failure.
