# Supplementary material for: Nut consumption and incidence of seven cardiovascular diseases
Source: Heart. 2018 Apr 16;104(19):1615–20. doi: 10.1136/heartjnl-2017-312819 (PMC6161661; doi:10.1136/heartjnl-2017-312819)
Supplement: Supplementary file 1 [file heartjnl-2017-312819supp001.docx]

**Supplementary material**

Nut consumption and incidence seven cardiovascular diseases

| **Table of contents** |  |
| --- | --- |
| **Figure S1.** Flow chart of study participants | **2** |
| **Figure S2.** Multivariable hazard ratios of nut consumption and cardiovascular disease stratified by sex, 1998-2014 | **3** |
| **Table S1.** Sensitivity analyses of nut consumption and cardiovascular disease excluding individuals with a history of diabetes, 1998-2014 | **4** |
| **Table S2.** Sensitivity analyses of nut consumption and cardiovascular disease with follow-up time restricted to 10 years, 1998-2007 | **5** |
| **Table S3** Sensitivity analyses of nut consumption and cardiovascular disease excluding the first two years of follow-up, 2000-2014 | **6** |

**Figure S1.** Flow chart of study participants

**Figure S2.** Multivariable hazard ratios* of nut consumption and cardiovascular disease stratified by sex, 1998-2014

*Hazard ratios were estimated by Cox proportional hazards models with age as the time scale and adjusted for education (less than high school, high school, or university), family history of myocardial infarction before 60 years of age (yes/no), smoking (never, past <20 pack-years, past ≥20 pack-years, current <20 pack-years, or current ≥20 pack-years), walking/bicycling (almost never, <20 min/day, 20–40 min/day, or >40 min/day), exercise (<1 hour/week, 1–2 hours/week, 3–4 hours/week, ≥5 hours/week), aspirin use (never, 1–6 tablets/week, or ≥7 tablets/week), and consumption of alcohol (never drinkers, past drinkers, or current drinkers of <1 drink/week, 1–6 drinks/week, 7–14 drinks/week, 14–21 drinks/week, or >21 drinks/week), fruits (quintiles), vegetables (quintiles), total energy intake (kcal/day; continuous), body mass index (<22.5, 22.5-24.9, 25.0-29.9, or ≥30.0 kg/m^2^), history of diabetes (yes/no), history of hypertension (yes/no), and history of hypercholesterolemia (yes/no). P-Interaction is the p-value for interaction by sex.

**Table S1.** Sensitivity analyses of nut consumption and cardiovascular disease excluding individuals with a history of diabetes, 1998-2014

|  | **Frequency of nut consumption** | | | |  |
| --- | --- | --- | --- | --- | --- |
| **Outcome** | **None** | **1–3/month** | **1–2/week** | **≥3/week** | **p Trend** |
| Myocardial infarction |  |  |  |  |  |
| Cases | 2623 | 1615 | 198 | 57 |  |
| Multivariable model 1* | 1.00 (reference) | 0.99 (0.93-1.05) | 0.92 (0.79-1.06) | 0.75 (0.58-0.98) | 0.02 |
| Multivariable model 2*^†^ | 1.00 (reference) | 1.00 (0.94-1.07) | 0.94 (0.81-1.09) | 0.77 (0.59-1.00) | 0.06 |
| Nonfatal myocardial infarction |  |  |  |  |  |
| Cases | 2098 | 1367 | 167 | 39 |  |
| Multivariable model 1* | 1.00 (reference) | 1.02 (0.96-1.10) | 0.95 (0.82-1.14) | 0.65 (0.49-0.92) | 0.03 |
| Multivariable model 2*^†^ | 1.00 (reference) | 1.03 (0.95-1.09) | 0.97 (0.81-1.11) | 0.67 (0.47-0.90) | 0.05 |
| Fatal myocardial infarction |  |  |  |  |  |
| Cases | 525 | 248 | 31 | 18 |  |
| Multivariable model 1* | 1.00 (reference) | 0.85 (0.73-0.99) | 0.81 (0.56-1.17) | 1.17 (0.72-1.88) | 0.67 |
| Multivariable model 2*^†^ | 1.00 (reference) | 0.86 (0.74-1.01) | 0.84 (0.58-1.22) | 1.17 (0.73-1.89) | 0.79 |
| Heart failure |  |  |  |  |  |
| Cases | 1821 | 870 | 110 | 55 |  |
| Multivariable model 1* | 1.00 (reference) | 0.84 (0.77-0.91) | 0.81 (0.66-0.98) | 0.92 (0.70-1.20) | 0.02 |
| Multivariable model 2*^†^ | 1.00 (reference) | 0.86 (0.79-0.93) | 0.84 (0.69-1.02) | 0.94 (0.72-1.24) | 0.06 |
| Atrial fibrillation |  |  |  |  |  |
| Cases | 4091 | 2610 | 312 | 104 |  |
| Multivariable model 1* | 1.00 (reference) | 0.95 (0.90-1.00) | 0.84 (0.75-0.95) | 0.77 (0.63-0.94) | <0.001 |
| Multivariable model 2*^†^ | 1.00 (reference) | 0.96 (0.92-1.01) | 0.87 (0.77-0.98) | 0.80 (0.66-0.97) | 0.002 |
| Aortic valve stenosis |  |  |  |  |  |
| Cases | 512 | 331 | 39 | 12 |  |
| Multivariable model 1* | 1.00 (reference) | 1.05 (0.91-1.21) | 0.96 (0.69-1.34) | 0.78 (0.44-1.38) | 0.52 |
| Multivariable model 2*^†^ | 1.00 (reference) | 1.07 (0.93-1.23) | 1.00 (0.72-1.39) | 0.81 (0.46-1.45) | 0.73 |
| Abdominal aortic aneurysm |  |  |  |  |  |
| Cases | 582 | 312 | 40 | 9 |  |
| Multivariable model 1* | 1.00 (reference) | 0.92 (0.79-1.05) | 0.89 (0.64-1.23) | 0.59 (0.30-1.14) | 0.06 |
| Multivariable model 2*^†^ | 1.00 (reference) | 0.93 (0.81-1.07) | 0.91 (0.66-1.26) | 0.61 (0.31-1.18) | 0.09 |
| Ischemic stroke |  |  |  |  |  |
| Cases | 1955 | 1239 | 172 | 55 |  |
| Multivariable model 1* | 1.00 (reference) | 1.04 (0.96-1.12) | 1.16 (0.99-1.36) | 0.97 (0.74-1.27) | 0.40 |
| Multivariable model 2*^†^ | 1.00 (reference) | 1.05 (0.97-1.13) | 1.18 (1.01-1.39) | 0.98 (0.74-1.28) | 0.28 |
| Intracerebral hemorrhage |  |  |  |  |  |
| Cases | 293 | 196 | 21 | 10 |  |
| Multivariable model 1* | 1.00 (reference) | 1.01 (0.84-1.22) | 0.82 (0.52-1.28) | 1.05 (0.56-1.99) | 0.80 |
| Multivariable model 2*^†^ | 1.00 (reference) | 1.02 (0.84-1.23) | 0.82 (0.52-1.29) | 1.04 (0.55-1.97) | 0.79 |

*Hazard ratios were estimated by Cox proportional hazards models with age as the time scale and stratified by sex, and adjusted for educational level (less than high school, high school, or university), family history of myocardial infarction before 60 years of age (yes/no), smoking (never, past <20 pack-years, past ≥20 pack-years, current <20 pack-years, or current ≥20 pack-years), walking/bicycling (almost never, <20 min/day, 20–40 min/day, or >40 min/day), exercise (<1 hour/week, 1–2 hours/week, 3–4 hours/week, or ≥5 hours/week), aspirin use (never, 1–6 tablets/week, or ≥7 tablets/week), and consumption of alcohol (never drinkers, past drinkers, or current drinkers of <1 drink/week, 1–6 drinks/week, 7–14 drinks/week, 14–21 drinks/week, or >21 drinks/week), fruits (quintiles), vegetables (quintiles), and total energy (kcal/day; continuous).

^†^Hazard ratios were adjusted for the same variables as in model 1 and further for potential intermediates of the nut-cardiovascular disease relationship, including body mass index (<22.5, 22.5-24.9, 25.0-29.9, or ≥30.0 kg/m^2^), history of diabetes (yes/no), history of hypertension (yes/no), and history of hypercholesterolemia (yes/no).

**Table S2.** Sensitivity analyses of nut consumption and cardiovascular disease with follow-up time restricted to 10 years, 1998-2007

|  | **Frequency of nut consumption** | | | |  |
| --- | --- | --- | --- | --- | --- |
| **Outcome** | **None** | **1–3/month** | **1–2/week** | **≥3/week** | **p Trend** |
| Myocardial infarction |  |  |  |  |  |
| Cases (n=2769) | 1700 | 926 | 106 | 38 |  |
| Multivariable model 1* | 1.00 (reference) | 0.95 (0.88-1.03) | 0.83 (0.68-1.01) | 0.80 (0.58-1.10) | 0.03 |
| Multivariable model 2*^†^ | 1.00 (reference) | 0.98 (0.90-1.06) | 0.86 (0.70-1.05) | 0.81 (0.59-1.12) | 0.07 |
| Nonfatal myocardial infarction |  |  |  |  |  |
| Cases | 1347 | 781 | 87 | 28 |  |
| Multivariable model 1* | 1.00 (reference) | 0.99 (0.90-1.08) | 0.84 (0.68-1.05) | 0.75 (0.52-1.10) | 0.06 |
| Multivariable model 2*^†^ | 1.00 (reference) | 1.02 (0.93-1.11) | 0.87 (0.70-1.09) | 0.77 (0.53-1.12) | 0.12 |
| Fatal myocardial infarction |  |  |  |  |  |
| Cases | 353 | 145 | 19 | 10 |  |
| Multivariable model 1* | 1.00 (reference) | 0.80 (0.66-0.98) | 0.79 (0.49-1.26) | 0.95 (0.50-1.78) | 0.29 |
| Multivariable model 2*^†^ | 1.00 (reference) | 0.82 (0.67-1.01) | 0.82 (0.51-1.31) | 0.97 (0.51-1.83) | 0.39 |
| Heart failure |  |  |  |  |  |
| Cases | 788 | 337 | 44 | 25 |  |
| Multivariable model 1* | 1.00 (reference) | 0.83 (0.73-0.95) | 0.84 (0.62-1.15) | 1.00 (0.67-1.50) | 0.31 |
| Multivariable model 2*^†^ | 1.00 (reference) | 0.87 (0.77-0.99) | 0.89 (0.66-1.22) | 1.02 (0.68-1.52) | 0.51 |
| Atrial fibrillation |  |  |  |  |  |
| Cases | 2071 | 1226 | 142 | 48 |  |
| Multivariable model 1* | 1.00 (reference) | 0.96 (0.89-1.03) | 0.83 (0.70-0.99) | 0.69 (0.52-0.93) | 0.001 |
| Multivariable model 2*^†^ | 1.00 (reference) | 0.98 (0.91-1.05) | 0.86 (0.72-1.02) | 0.72 (0.54-0.96) | 0.008 |
| Aortic valve stenosis |  |  |  |  |  |
| Cases | 252 | 143 | 12 | 6 |  |
| Multivariable model 1* | 1.00 (reference) | 1.01 (0.81-1.24) | 0.65 (0.36-1.17) | 0.80 (0.35-1.80) | 0.28 |
| Multivariable model 2*^†^ | 1.00 (reference) | 1.03 (0.83-1.28) | 0.68 (0.38-1.22) | 0.83 (0.37-1.89) | 0.39 |
| Abdominal aortic aneurysm |  |  |  |  |  |
| Cases | 244 | 113 | 16 | 2 |  |
| Multivariable model 1* | 1.00 (reference) | 0.94 (0.75-1.18) | 1.04 (0.62-1.75) | 0.31 (0.08-1.27) | 0.16 |
| Multivariable model 2*^†^ | 1.00 (reference) | 0.95 (0.75-1.19) | 1.06 (0.64-1.78) | 0.33 (0.08-1.32) | 0.19 |
| Ischemic stroke |  |  |  |  |  |
| Cases | 1120 | 662 | 100 | 34 |  |
| Multivariable model 1* | 1.00 (reference) | 1.07 (0.97-1.18) | 1.31 (1.06-1.61) | 1.08 (0.76-1.52) | 0.08 |
| Multivariable model 2*^†^ | 1.00 (reference) | 1.11 (1.01-1.23) | 1.36 (1.10-1.67) | 1.10 (0.78-1.55) | 0.03 |
| Intracerebral hemorrhage |  |  |  |  |  |
| Cases | 181 | 102 | 8 | 6 |  |
| Multivariable model 1* | 1.00 (reference) | 0.95 (0.74-1.22) | 0.59 (0.29-1.22) | 1.14 (0.50-2.60) | 0.62 |
| Multivariable model 2*^†^ | 1.00 (reference) | 0.95 (0.74-1.22) | 0.59 (0.29-1.21) | 1.13 (0.49-2.57) | 0.61 |

*Hazard ratios were estimated by Cox proportional hazards models with age as the time scale and stratified by sex, and adjusted for educational level (less than high school, high school, or university), family history of myocardial infarction before 60 years of age (yes/no), smoking (never, past <20 pack-years, past ≥20 pack-years, current <20 pack-years, or current ≥20 pack-years), walking/bicycling (almost never, <20 min/day, 20–40 min/day, or >40 min/day), exercise (<1 hour/week, 1–2 hours/week, 3–4 hours/week, or ≥5 hours/week), aspirin use (never, 1–6 tablets/week, or ≥7 tablets/week), and consumption of alcohol (never drinkers, past drinkers, or current drinkers of <1 drink/week, 1–6 drinks/week, 7–14 drinks/week, 14–21 drinks/week, or >21 drinks/week), fruits (quintiles), vegetables (quintiles), and total energy (kcal/day; continuous).

^†^Hazard ratios were adjusted for the same variables as in model 1 and further for potential intermediates of the nut-cardiovascular disease relationship, including body mass index (<22.5, 22.5-24.9, 25.0-29.9, or ≥30.0 kg/m^2^), history of diabetes (yes/no), history of hypertension (yes/no), and history of hypercholesterolemia (yes/no).

**Table S3.** Sensitivity analyses of nut consumption and cardiovascular disease excluding the first two years of follow-up, 2000-2014

|  | **Frequency of nut consumption** | | | |  |
| --- | --- | --- | --- | --- | --- |
| **Outcome** | **None** | **1–3/month** | **1–2/week** | **≥3/week** | **p Trend** |
| Myocardial infarction |  |  |  |  |  |
| Cases (n=4329) | 2553 | 1526 | 185 | 65 |  |
| Multivariable model 1* | 1.00 (reference) | 0.96 (0.90-1.03) | 0.89 (0.76-1.03) | 0.87 (0.68-1.11) | 0.06 |
| Multivariable model 2*^†^ | 1.00 (reference) | 0.99 (0.92-1.05) | 0.90 (0.79-1.07) | 0.88 (0.69-1.13) | 0.16 |
| Nonfatal myocardial infarction |  |  |  |  |  |
| Cases (n=3533) | 2044 | 1282 | 162 | 45 |  |
| Multivariable model 1* | 1.00 (reference) | 0.98 (0.91-1.06) | 0.95 (0.81-1.12) | 0.75 (0.56-1.02) | 0.07 |
| Multivariable model 2*^†^ | 1.00 (reference) | 1.01 (0.94-1.08) | 0.97 (0.83-1.15) | 0.77 (0.57-1.03) | 0.14 |
| Fatal myocardial infarction |  |  |  |  |  |
| Cases (n=796) | 509 | 244 | 23 | 20 |  |
| Multivariable model 1* | 1.00 (reference) | 0.86 (0.74-1.01) | 0.62 (0.41-0.95) | 1.32 (0.84-2.08) | 0.69 |
| Multivariable model 2*^†^ | 1.00 (reference) | 0.89 (0.76-1.05) | 0.65 (0.42-0.99) | 1.34 (0.85-2.11) | 0.88 |
| Heart failure |  |  |  |  |  |
| Cases (n=2811) | 3078 | 1557 | 177 | 92 |  |
| Multivariable model 1* | 1.00 (reference) | 0.84 (0.78-0.92) | 0.79 (0.65-0.96) | 0.94 (0.72-1.23) | 0.03 |
| Multivariable model 2*^†^ | 1.00 (reference) | 0.88 (0.81-0.95) | 0.83 (0.68-1.01) | 0.98 (0.75-1.28) | 0.12 |
| Atrial fibrillation |  |  |  |  |  |
| Cases (n=6902) | 3970 | 2521 | 305 | 106 |  |
| Multivariable model 1* | 1.00 (reference) | 0.96 (0.90-1.00) | 0.86 (0.76-0.96) | 0.80 (0.66-0.97) | 0.001 |
| Multivariable model 2*^†^ | 1.00 (reference) | 0.97 (0.92-1.02) | 0.89 (0.79-1.00) | 0.84 (0.69-1.02) | 0.008 |
| Aortic valve stenosis |  |  |  |  |  |
| Cases (n=910) | 513 | 345 | 38 | 14 |  |
| Multivariable model 1* | 1.00 (reference) | 1.11 (0.96-1.27) | 0.94 (0.67-1.1 | 0.88 (0.51-1.50) | 0.80 |
| Multivariable model 2*^†^ | 1.00 (reference) | 1.14 (0.99-1.31) | 0.99 (0.71-1.38) | 0.82 (0.54-1.58) | 0.91 |
| Abdominal aortic aneurysm |  |  |  |  |  |
| Cases (n=908) | 553 | 306 | 40 | 9 |  |
| Multivariable model 1* | 1.00 (reference) | 0.95 (0.82-1.09) | 0.92 (0.66-1.27) | 0.61 (0.31-1.18) | 0.12 |
| Multivariable model 2*^†^ | 1.00 (reference) | 0.96 (0.83-1.11) | 0.94 (0.68-1.31) | 0.64 (0.33-1.24) | 0.18 |
| Ischemic stroke |  |  |  |  |  |
| Cases (n=3410) | 1974 | 1214 | 161 | 61 |  |
| Multivariable model 1* | 1.00 (reference) | 1.01 (0.94-1.09) | 1.07 (0.91-1.26) | 1.05 (0.81-1.36) | 0.49 |
| Multivariable model 2*^†^ | 1.00 (reference) | 1.04 (0.96-1.12) | 1.10 (0.93-1.30) | 1.06 (0.82-1.37) | 0.28 |
| Intracerebral hemorrhage |  |  |  |  |  |
| Cases (n=468) | 257 | 182 | 20 | 9 |  |
| Multivariable model 1* | 1.00 (reference) | 1.07 (0.88-1.31) | 0.88 (0.55-1.39) | 1.05 (0.54-2.06) | 0.99 |
| Multivariable model 2*^†^ | 1.00 (reference) | 1.07 (0.88-1.30) | 0.88 (0.55-1.39) | 1.04 (0.53-2.04) | 0.97 |

*Hazard ratios were estimated by Cox proportional hazards models with age as the time scale and stratified by sex, and adjusted for educational level (less than high school, high school, or university), family history of myocardial infarction before 60 years of age (yes/no), smoking (never, past <20 pack-years, past ≥20 pack-years, current <20 pack-years, or current ≥20 pack-years), walking/bicycling (almost never, <20 min/day, 20–40 min/day, or >40 min/day), exercise (<1 hour/week, 1–2 hours/week, 3–4 hours/week, or ≥5 hours/week), aspirin use (never, 1–6 tablets/week, or ≥7 tablets/week), and consumption of alcohol (never drinkers, past drinkers, or current drinkers of <1 drink/week, 1–6 drinks/week, 7–14 drinks/week, 14–21 drinks/week, or >21 drinks/week), fruits (quintiles), vegetables (quintiles), and total energy (kcal/day; continuous).

^†^Hazard ratios were adjusted for the same variables as in model 1 and further for potential intermediates of the nut-cardiovascular disease relationship, including body mass index (<22.5, 22.5-24.9, 25.0-29.9, or ≥30.0 kg/m^2^), history of diabetes (yes/no), history of hypertension (yes/no), and history of hypercholesterolemia (yes/no).
